# *Astragalus* injection ameliorates lipopolysaccharide-induced cognitive decline via relieving acute neuroinflammation and BBB damage and upregulating the BDNF-CREB pathway in mice

**DOI:** 10.1080/13880209.2022.2062005

**Published:** 2022-05-19

**Authors:** Ke Liu, Guoran Wan, Ruhong Jiang, Li Zou, Dong Wan, Huifeng Zhu, Shan Feng

**Affiliations:** a Department of Traditional Chinese Medicine, College of Pharmaceutical Sciences and Traditional Chinese Medicine, Southwest University, Chongqing, China; b Department of Emergency & Critical Care Medicine, The First Affiliated Hospital of Chongqing Medical University, Chongqing, China; c Department of Psychiatry, First Clinical College of Chongqing Medical University, Chongqing, China; d Department of General Practice, Fifth Clinical College of Chongqing Medical University, Chongqing, China

**Keywords:** Astragaloside IV, TrkB, neurodegeneration, tight junction, post-sepsis cognitive impairment

## Abstract

**Context:**

Post-sepsis cognitive impairment is one of the major sequelae observed in survivors of sepsis. *Astragalus* injection is the normally preferred treatment in sepsis in clinical settings.

**Objective:**

This study evaluated the benefits and related mechanism of *Astragalus* injection on post-sepsis cognitive impairment.

**Materials and methods:**

C57BL/6J mice were divided into three groups: Control, LPS (2.5 mg/kg, i.p.), and LPS **+** *Astragalus* injection (5.0 mL/kg). The surviving mice from sepsis were injected with material named *Astragalus* injection continuously for 13 days. Behavioural tests were first conducted to evaluate the benefits. Second, inflammatory cytokines secretion, BBB integrity, neurodegeneration, and protein expression was evaluated *in vivo* and *in vitro*.

**Results:**

Compared with the LPS group, mice in *Astragalus* injection group exhibited shorter escape latency (34.6 s versus 24.5 s) in the Morris water maze test. Treatment with *Astragalus* injection could reverse LPS-induced neuroinflammation in mice and BV2 cells. Continuous *Astragalus* injection treatment not only prevented blood–brain barrier dysfunction, but also prevented neurodegeneration. Further molecular docking tests and western blot results reflected that the main constituents of *Astragalus* injection could interact with TrkB (the estimated binding energy values were −7.0 to −5.0 kcal/mol) and upregulate the protein expression of BDNF/TrkB/CREB signalling pathway during the chronic stage in mice.

**Discussion:**

*Astragalus* injection treatment could reduce neuroinflammation, reverse BBB dysfunction, prevent neurodegeneration, and upregulate BDNF-CREB pathway during LPS-induced sepsis, ultimately preventing the development of cognitive decline.

**Conclusion:**

*Astragalus* injection could be a potential preventive and therapeutic strategy for sepsis survivors in clinical settings.

## Introduction

Sepsis is a serious complication of infection, which can lead to tissue damage, organ failure, and death. A precise estimate of the global epidemiological burden of sepsis is difficult to ascertain. Some studies showed that more than 30 million people worldwide suffered from it every year, potentially leading to 6 million deaths (Fleischmann et al. 2016). However, the effects of sepsis do not end at hospital discharge. According to the Global Sepsis Alliance, many sepsis survivors suffer from the consequences of sepsis for the rest of their lives, such as sadness, difficulty sleeping, poor memory, difficulty concentrating, fatigue, and anxiety (https://www.global-sepsis-alliance.org/sepsis). Despite major concerns in the diagnosis and clinical management of sepsis, strategies for the treatment of related sequelae are still missing.

Post-sepsis cognitive impairment is one of the major consequences observed in survivors of sepsis. Neuroinflammation, oxidative damage, and vascular permeability are the main causes of brain damage during systemic inflammation (Henry et al. 2009; Michels et al. 2014; Schwalm et al. 2014; Danielski et al. 2018; Hoogland et al. 2018). Lipopolysaccharide (LPS)-induced rodent model of sepsis is always used in the study of septic encephalopathy. By using IL-1RI^−/−^ mice, researchers demonstrated that interleukin-1 (IL-1) mainly mediates the LPS-induced depressive effects and tumour necrosis factor-α (TNF-α) simply replaces IL-1 when the last cytokine is deficient (Bluthé et al. 2000). A similar study found that peripheral LPS challenge could promote microglial activation, subsequently releasing pro-inflammatory cytokines and ultimately resulting in neuronal injury (Matt et al. 2016). In addition, the blood–brain barrier (BBB) is also important for the maintenance of brain homeostasis. Peripheral production of pro-inflammatory cytokines and reactive oxygen species directly damages BBB, which in turn allows the transportation of cytotoxic mediators to the central nervous system, thus starting a vicious circle (Nishioku et al. 2009; Banks et al. 2015; Chi et al. 2019). Taken together, keeping the integrality of BBB and inhibiting neuroinflammation in the brain are important in alleviating septic encephalopathy.

Astragali Radix is the dried root of *Astragalus membranaceus* (Fisch.) Bge. var. *mongholicus* (Bge.) Hsiao (Fabaceae). It is used to supplement qi, outthrust toxins, expel pus, close sores, and engender flesh according to the Chinese medicine theory. *Astragalus* injection is the extract obtained from Astragali Radix by water extraction and alcohol precipitation method. It is also listed in the Chinese Pharmacopoeia (2020 edition) with stipulation that the astragaloside IV in this product should not be less than 0.08 mg/mL (1 mL *Astragalus* injection equals 2 g dried Astragali Radix crude drugs). Additionally, *Astragalus* injection, with flavonoid, isoflavones, saponin glycosides, and polysaccharoses as main active constituents, is normally used in viral myocarditis, viral enteritis, diabetic nephropathy, hepatitis, and sepsis in China (Chen 2008; Wu 2013; Yu et al. 2019). For sepsis survivors, *Astragalus* injection could improve patient immunity, such as controlling the levels of CD4/CD8 and inflammatory cytokines (Wu 2013). Animal studies further demonstrated that *Astragalus* injection or its active compounds (astragaloside IV, polysaccharides) could inhibit LPS-induced inflammation and nuclear factor kappa-B (NF-κB) activation in mouse macrophages and increase the integrity of endothelial cells (Yang et al. 2013; Li et al. 2017; Leng et al. 2018). However, whether it can ameliorate the post-sepsis cognitive impairment is not known.

Brain-derived neurotrophic factor (BDNF) is a major regulator of neural stem cell survival and differentiation, axon/dendrite differentiation (Heldt et al. 2007), synapse formation and maturation, and refinement of developing neural circuits. TrkB is a neurotrophin receptor that binds to BDNF, and one of its downstream signalling transduction pathway is CaMKIIα/CREB (Kozisek et al., 2008; Tang et al., 2019; Esvald et al., 2020). Meanwhile, previous studies showed that Astragali Radix polysaccharides could improve the impaired learning and memory functions in aged rats via upregulating the activity of hippocampal cyclic adenosine monophosphate (cAMP) response element–binding protein (CREB)/BDNF cascade (Yao et al. 2014). Additionally, the present molecular docking test results also showed that four main active components (astragaloside IV, formononetin, formononetin-7-*O*-glucoside and calycosin) in *Astragalus* injection could interact with TrkB.

Most importantly, the present study was first performed to evaluate the beneficial effects of *Astragalus* injection on post-sepsis cognitive decline in an LPS-induced mouse model of sepsis. Then, inflammatory cytokines secretion analysis, BBB integrity and neurodegeneration determination, molecular docking tests, as well as the detection of the protein expression of the pathway of BDNF/TrkB/CREB were conducted to explore the related mechanism of action.

## Materials and methods

### Materials

*Astragalus* injection was purchased from Chia Tai Tianqing Pharmaceutical Group Co., Ltd. (Hangzhou, China). LPS (from *Escherichia coli* O111:B4) was purchased from Sigma (St. Louis, MO). Enzyme-linked immunosorbent assay (ELISA) kits for IL-1β, TNF-α, and IL-6 were purchased from Boster Biological Technology (Wuhan, China). Rabbit polyclonal antibodies against zonula occludens-1 (ZO-1), clauidn-5, and occludin were purchased from Cell Signaling Technology Biotechnology (Boston, MA). DyLight 649 (or DyLight 488)-conjugated donkey anti-rabbit or anti-goat antibodies were purchased from Boster Biological Technology (Wuhan, China). BV2 cell line (Passage 4) was purchased from Shanghai Guandao Bioengineering Co., Ltd. (Shanghai, China).

#### Chemical analysis of *Astragalus* injection

The content of astragaloside IV, calycosin, calycosin-7-*O*-glucoside, formononetin, fomononetin-7-*O*-glucoside in *Astragalus* injection were preliminarily analyzed using the Waters Acquity UPLC Sample Manager and a Waters Acquity UPLC Binary Solvent Manager connected to a Waters XEVO-G2QTOF mass spectrometer equipped with a combined electrospray ionization (ESI) probe and Mass Lynx 4.1 software (Waters, MA). An Acquity UPLC BEH C18 column (1.8 µm, 2.1 × 100 mm) was used. The instrument settings were as follows: ESI+; source temperature, 110 °C; desolvation temperature, 450 °C; capillary voltage, 3.0 kV; desolvation N_2_, 800 L/h; cone N_2_, 50 L/h; collision energy, 2 eV for astragaloside IV, 24 eV for calycosin, 18 eV for calycosin-7-*O*-glucoside, 40 eV for formononetin and 16 eV for fmononetin-7-*O*-glucoside; cone voltage, 100 eV for astragaloside IV, 52 eV for calycosin, 30 eV for calycosin-7-*O*-glucoside, 54 eV for formononetin and 28 eV for fomononetin-7-*O*-glucoside. Multiple reaction monitoring (MRM) mode was chosen for each analyte. The first parent/daughter ion pair of astragaloside IV, calycosin, calycosin-7-*O*-glucoside, formononetin, fomononetin-7-*O*-glucoside were *m*/*z* 807.5→807.5, 285.0 → 269.9, 447.1 → 285.0, 269.0 → 197.0, 431.2 → 269.0, respectively. The solvent system consisted of solvent A (0.1%FA in water) and solvent B (0.1% FA in acetonitrile). The gradient was as follows: 0–15 min, from 2% to 100% B; 15.0–20.0 min, from 100% to 100%B, and then back to 2% B at 5.0 min for column re-equilibrium prior to the next injection. The flow rate of mobile phase was 0.3 mL/min. The injection volume was 1 µL and the cycle time was 20 min/injection.

#### Animal treatments

C57BL/6J male mice were purchased from the Experimental Animal Centre of Chongqing Medical University (Certificate number SYXK, 2012-0001). All mice were housed and bred in a climate-controlled environment at the animal facility of Southwest University. All animal experiments were conducted in accordance with the National Institutes of Health (NIH) guidelines for the care and use of laboratory animals (NIH Publications No. 8023), and approved by experimental animal ethics committee of School of Pharmaceutical Sciences & School of Chinese Medicine, Southwest University (approval number yxy202116).

**Figure 1.**
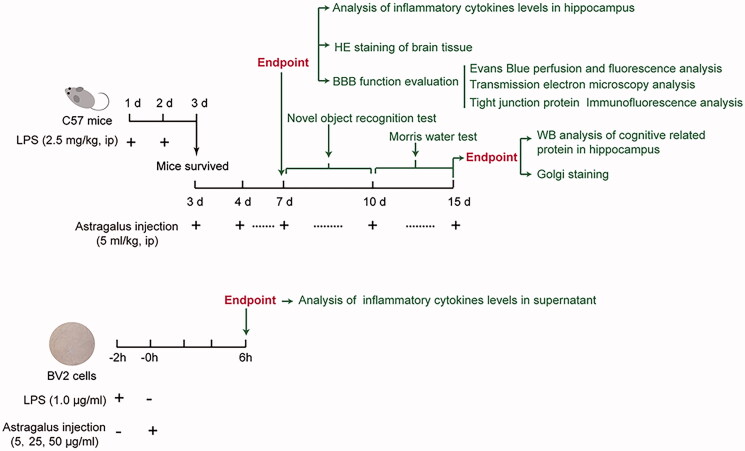
Scheme of the study. *Astragalus* injection ameliorated lipopolysaccaride-induced cognitive decline and blood–brain barrier permeability disruption *in vivo* (top) and *in vitro* (bottom).

The scheme of the study is shown in Figure 1. The dosage of *Astragalus* injection (5.0 mL/kg) applied in *in vivo* experiments was calculated from its clinical usage (20–30 mL per day). Besides, the LPS dosing schedule was referenced from previous studies (Nishioku et al. 2009; Banks et al. 2015; Chi et al. 2019).

For *in vivo* experiments, 60 C57BL/6J mice were divided into three groups: Control group, LPS group and *Astragalus* injection group (*n* = 20 in each group). Mice in LPS and *Astragalus* injection groups were injected with LPS (2.5 mg/kg, i.p.) continuously for 2 subsequent days. On the third day, the surviving mice in *Astragalus* injection group were injected with 5.0 mL/kg *Astragalus* injection (i.p., once a day) continuously for 13 days. In the control groups, the *Astragalus* injection or LPS was replaced with saline. On the 7th day, 10 mice in each group were euthanized and the blood and brain were extracted for further analysis. For the rest of 10 mice in each group, the novel object recognition test and Morris water maze test were conducted from the 7th to 15th days. Finally, the mice were euthanized, and brains were isolated for further analysis when behavioural tests were completed.

#### BV2 cell treatments

BV2 microglial cells were purchased from Shanghai GuanDao Biological Engineering Co., Ltd., and incubated in DMEM medium with 10% FBS at 37 °C and 5% CO_2_. For *in vitro* experiments (Figure 1), each group was assigned with 6 wells and cells at a density of 5 × 10^5^ cells per well. Cells were seeded in 6 well plates at a volume of 1 mL/well. When the confluence reached 80% (24 h later), *Astragalus* injection (5, 25, and 50 μg/mL) was added directly to the culture medium after pre-incubating LPS (1 μg/mL) with BV2 cells for 2 h. For the analysis of IL-1β, TNF-α, and IL-6 secretion, the cell culture medium was collected 6 h after adding LPS.

#### Novel object recognition test

On days 7 and 8, the novel object recognition test was conducted in mice from each group as previously described (Tang et al. 2019). Briefly, the mice were individually placed in an empty open field for 5 min to explore and be familiar with the open field the day before the test. Two identical Falcon tissue culture flasks were placed 5 cm away from the walls in the open field 24 h later. Then, the mice were individually placed in an open field, with head positioned opposite to the objects, and were allowed to explore freely for 10 min. After the familiarization session, the mice were returned to their home cages. The objects and the open field were cleaned with 70% ethanol. Six hours later, a novel object (Lego brick) was used to replace one of the two identical objects for the test session. The two objects were randomly placed at the same locations as in the familiarization session, and mice were allowed to explore freely for 10 min. Other procedures were not changed.

All experimental sessions were recorded by a video camera. According to previous report (Leger et al. 2013), a second stopwatch was used to record the time spent exploring each object until 20 s of total exploration time has been reached. Object exploration was scored whenever the mouse sniffed the object or touched the object while looking at it (i.e., when the distance between the nose and the object was less than 2 cm). Climbing onto the object (unless the mouse sniffs the object it has climbed on) or chewing the object does not qualify as exploration. The scoring was conducted independently by two experimenters, and the average exploration time was used in the analysis. The animal's scoring will be excluded from the data analysis, if the total exploration time was less than 20 s.

#### Morris water maze test

From the 10th day, all mice were subjected to the Morris water maze test. A previously described protocol (Tang et al. 2019) was used. First, 5 days of learning trials were performed, with four trials per day and 15 s of interval between trials. The platform was located in the southwest (SW) quadrant, 1 cm lower than the water level. Distal cues were available for assisting the mice in navigating to the hidden platform. A set of semi-randomly start positions was applied. If a mouse failed to find the platform within 60 s, it was picked up and placed on the platform for 15 s. The time spent in finding the hidden platform was recorded as escape latency. Twenty-four hours after the last learning trial, a probe trial was administered to assess the reference memory of the mice. The platform was removed in this trial. The mice were allowed to swim for 60 s in the pool, with the northwest (NE) quadrant as the start position. The crossing numbers on the location of the removed platform and the time spent in the platform quadrant were recorded. Table 1 presents the start positions for the mice in the learning trials and the probe trial (Vorhees and Williams 2006). All experimental sessions were recorded by a video camera. The above parameters were later obtained using Noldus EthoVision XT 8.5 (Wageningen, The Netherlands).

**Table 1.** Morris water maze spatial (hidden platform) start positions.

Acquisition				
Day	Trial 1	Trial 2	Trial 3	Trial 4
1	N	E	SE	NW
2	SE	N	NW	E
3	NW	SE	E	N
4	E	NW	N	SE
5	N	SE	E	NW
6 (Probe)	NE			

N: north; E: east; SE: south east; NW: north west; NE, north east. Referenced from Vorhees and Williams (2006).

#### Molecular docking study of *Astragalus* injection components and TrkB

Molecular docking studies were performed to investigate the binding mode between the *Mus musculus* TrkB and the test compounds (astragaloside IV, calycosin, calycosin-7-*O*-glucoside, formononetin) using Autodock vina 1.1.2 (Trott and Olson 2010). We applied human TrkA (PDB ID: 5KML) as the template for the *Mus musculus* TrkB, and the 3D structure of *Mus musculus* TrkB was built by SWISS-MODEL, a fully automated protein structure homology-modelling server. The 2D structure of the compound was drawn by ChemBioDraw Ultra 14.0 and was optimized by MM2 method using ChemBio3D Ultra 14.0 software to obtain the 3D structure. The AutoDockTools 1.5.6 package (Sanner 1999; Morris et al. 2009) was employed to generate the docking input files. During the molecular docking study, the polar hydrogen atoms and Kollman united atom type charge were added to the protein. The search grid of the TrkB was identified as centre_*x*: −16.679, centre_*y*: −4.877, and centre_*z*: −23.757 with dimensions size_*x*: 15, size_*y*: 15, and size_*z*: 15. The value of exhaustiveness was set to 20. For Vina docking, the default parameters were used if it was not mentioned. The best-scoring pose as judged by the Vina docking score was chosen and visually analyzed using PyMoL 1.7.6 software (www.pymol.org).

#### Measurement of the levels of IL-1β, TNF-α, and IL-6 using ELISA

The levels of IL-1β, TNF-α, and IL-6 in the blood, hippocampus, and culture medium of BV2 cells were measured using commercially available ELISA kits. All incubation and washing steps were performed following the manufacturer’s recommended protocols. The optical density values were measured using a FlexStation 3 instrument. The cytokine levels were determined by comparison with the standard curve.

#### Haematoxylin and eosin staining

The mouse brains were fixed in neutral buffered formalin for 48 h, embedded in paraffin, cut into 5 μm thick sections, and stained with haematoxylin and eosin according to a standard protocol.

#### Evans blue perfusion analysis

Evans blue perfusion analysis was performed as described previously (Feng et al. 2018). The mice were anaesthetized with 10% chloral hydrate (0.1 mL/10 g, ip) and intracardially perfused with prewarmed 0.9% NaCl briefly to wash out blood cells, followed by 0.5% Evans blue in cold 4% paraformaldehyde. After perfusion, the brains were taken out, post-fixed in 4% paraformaldehyde at 4 °C for 4 h, and then cryoprotected in 20% and 30% sucrose solutions in phosphate-buffered saline (PBS) at 4 °C for 3 days. The brain tissues were cut into 30 μm thick sections in a cryostat (Leica Microsystems, Wetzlar, Germany). The sections were mounted on gel-coated slides, dried at 37 °C for 1 h, and kept at −20 °C for use. The Evans blue–perfused cerebral microvessels in the sections, which exhibited fluorescence when Evans blue bound to proteins, were examined and images were captured using a Leica fluorescence microscope equipped with a CCD camera (Leica Microsystems).

#### Transmission electron microscopic analysis of tight junction

Transmission electron microscopic analysis was performed as described previously (Feng et al. 2018). The mice in each group were anaesthetized with 10% chloral hydrate (0.1 mL/10 g, i.p.) and perfused transcardially with 2.5% glutaraldehyde in 0.1 M PBS. Approximately 1 mm^3^ of the brain tissue was taken and fixed in freshly prepared 3% glutaraldehyde for 4 h at 4 °C and post-fixed in 1% osmium tetroxide for 2 h. The specimens were then dehydrated through a graded series of ethanol concentrations (50% → 70% → 80% → 90% → 95% → 100%), and embedded in Epon 812 overnight. Then, 60–80 nm ultrathin brain sections were obtained and stained with uranyl acetate and lead citrate. The sections were then examined under a transmission electron microscope (Hitachi-H7500, Tokyo, Japan).

#### Immunohistochemistry

The mouse brains were fixed in freshly prepared 4% paraformaldehyde in PBS (pH = 7.4) for 24 h at 4 °C. They were then transferred to 20% sucrose–PBS (pH = 7.4) until the tissues submerged into the solution (about 24–36 h). The brains were then mounted in the mounting medium and frozen at −80 °C. The OTC-embedded brains were cut into 10 μm sections using a cryostat, and the sections were picked up with SuperFrost/Plus slides. Later, the sections were blocked in blocking buffer (0.3% Triton X-100 in PBS) at room temperature for 2 h. After that, primary antibodies against CD31 (1:100), ZO-1 (1:50), clauidn-5 (1:100), and occludin (1:100) were incubated with the sections overnight at 4 °C. Dylight-488-conjugated donkey anti-rabbit and Dylight-649-conjugated donkey anti-goat secondary antibodies were then incubated with the sections in the dark for 1 h at room temperature. Fluorescent images were acquired using a Leica Sp2 confocal microscope.

#### Golgi staining

Three mouse brains per group were stained using a Golgi-staining kit following the manufacturer’s protocol (Servicebio, Wuhan, China). The brain tissues were submerged in Gorky's staining solution completely in a cool and ventilated place to avoid light for 14 days (the new staining solution was changed after the first 48 h of immersion and then changed once every 3 days for a total of 14 days). The tissues were then transferred to 15% sucrose–PBS (pH = 7.4) and dehydrated in the dark for 1 day at 4 °C. Later, the tissue was transferred to 30% sucrose–PBS (pH = 7.4) and further dehydrated in the dark for 2 days at 4 °C. Next, the tissues were washed with distilled water for 1 min and then immersed in concentrated ammonia water for 45 min. Then, the tissues were washed again with distilled water for 1 min and immersed in an acid film fixer for 45 min. The Golgi-stained brains were sectioned to 100 μm using a frozen microtome (Thermo, CryoStar NX50). The sections were visualized under an upright microscope (Nikon Eclipse E100 microscope). For spine analysis, the dendrites in 20–30 μm lengths of the three tertiary segments were used to measure dendritic spine density via ImageJ (NIH Image J system, Bethesda, MD).

#### Western blot analysis

After behavioural tests, the mice were sacrificed, brains were collected, and hippocampi were separated. The hippocampi were homogenized in RIPA lysis buffer and centrifuged at 12,000 *g* for 15 min (4 °C). The supernatants were collected and used for Western blot analysis. Total protein concentrations were measured by the BCA protein assay method. Then, 30 μg of total proteins was loaded onto SDS-PAGE gels after added 5× sample buffer and boiled at 95 °C for 10 min. The separated proteins were then transferred to PVDF membranes and blocked with 5% non-fat milk for 1 h at room temperature. The PVDF membranes with proteins were incubated with diluted primary antibodies at 4 °C overnight, including anti-TrkB (1:200), anti-p-TrkB(Y516) (1:200), anti-CaMKIIα (1:200), anti-p-CaMKIIα (Thr286, 1:200), anti-CREB1 (1:200), anti-p-CREB1 (Ser133) (1:500), anti-BDNF (1:500), and anti-β-tubulin (1:2000) antibodies. After washing with TBST three times, the membranes were incubated with relative sources of secondary antibodies (1:10,000) at room temperature for 2 h. Finally, the protein bands were detected with the Immobilon Western Chemiluminescent HRP Substrate (Millipore Sigma, Billerica, MA). The images were analyzed with Image J. The signals of protein targets were normalized to β-actin.

#### Data statistics

The data were expressed as mean ± standard deviation and analyzed using SPSS version 19.0 statistical software (SPSS, Chicago, IL). Comparisons between multiple groups were performed using one-way analysis of variance (ANOVA) followed by Tukey’s comparison test. The escape latency in the Morris water maze test was analyzed with repeated-measure ANOVA, followed by Tukey’s comparison test. The exploration time in the novel object recognition test, the platform crossing numbers, and the time spent in platform quadrant in the Morris water maze test were analyzed with the Kruskal–Wallis test followed by the Mann–Whitney *U* test*. p* Values less than 0.05 were considered significant.

## Results

### Chemical analysis of *Astragalus* injection

**Figure 2.**
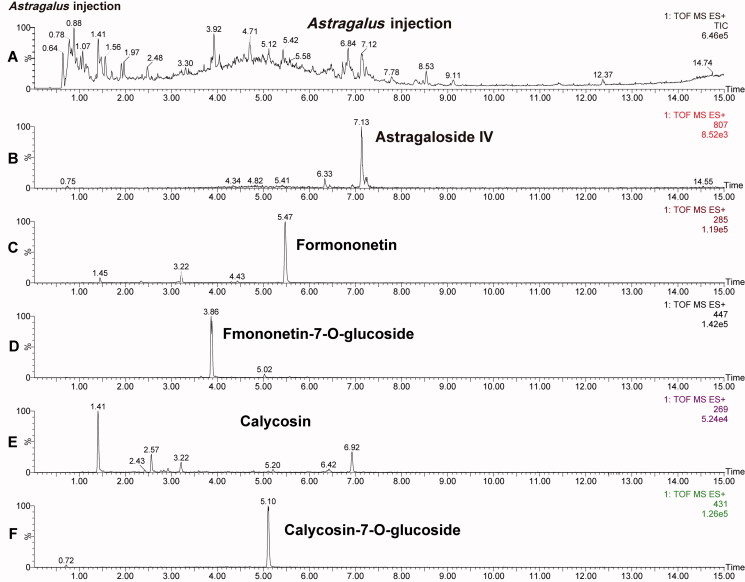
UPLC-ESI-MS/MS total ion current chromatograms of *Astragalus* injection and related extracted ion chromatograms. (A) Total ion current chromatograms of mixed standard. (B–F) The extracted ion chromatogram of astragaloside IV, formononetin, fomononetin-7-*O*-glucoside, calycosin and calycosin-7-*O*-glucoside, respectively.

To evaluate the contents of main active components in *Astragalus* injection. Astragaloside IV, calycosin, calycosin-7-*O*-glucoside, formononetin, fomononetin-7-*O*-glucoside in *Astragalus* injection were analyzed via UPLC-MS/MS method (Figure 2).

Analysis results showed that the content of astragaloside IV, calycosin, calycosin-7-*O*-glucoside, formononetin, fomononetin-7-*O*-glucoside in *Astragalus* injection was 435.0, 16.2, 67.0, 0.34, and 25.9 µg/mL, respectively. Meanwhile, the content of astragaloside IV (435.0 µg/mL) meets the requirement of the Chinese Pharmacopoeia (2020 edition: The astragaloside IV in *Astragalus* injection should not be less than 80 µg/mL).

### *Astragalus* injection improved recognition memory in mice

**Figure 3.**
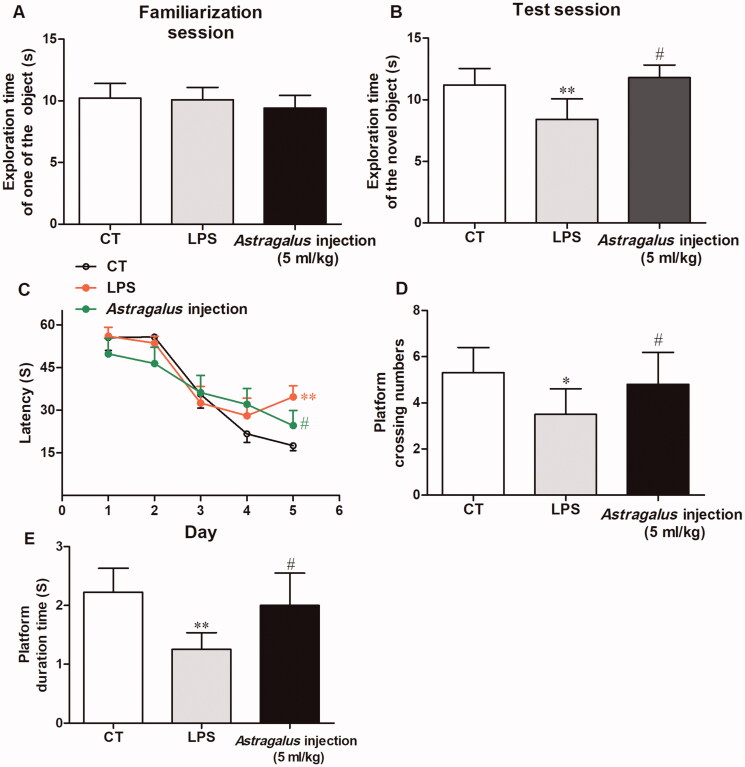
Effects of *Astragalus* injection in improving mice recognition memory shown by the novel object recognition test (A and B) and Morris water maze test (C, D, and E). (A) Exploration time of one of the two identical objects in the familiarization session; (B) exploration time of the novel object in the test session; (C) escape latency in the 5-day acquisition trials; (D) platform crossing numbers in the probe trial; and (E) time spent in platform quadrant in the probe trial. Values are presented as means ± SD (*n* = 10). **p < 0.01 versus the LPS group; ^#^*p* < 0.05, versus the model group. CT: control, LPS: lipopolysaccharide.

The behavioural test results showed that the mice in the LPS group had impaired cognitive function, and the treatment with *Astragalus* injection ameliorated this impairment. The results of the novel object recognition test showed that the exploring time of one of the two same objects had no difference in the familiarization session (Figure 3(A)). However, during the test session, the LPS-treated mice spent shorter time in exploring the novel object compared with the vehicle-treated mice (*p* < 0.01, Figure 3(B)). The mice in the *Astragalus* injection group spent more time in exploring the novel object compared with the LPS-treated mice (*p* < 0.05, Figure 3(B)).

Morris water maze test results showed that the mice in the LPS group had longer escape latency compared with the vehicle-treated mice on the fifth day during the 5-day spatial learning period (*p* < 0.01, Figure 3(C)). Meanwhile, the mice in the *Astragalus* injection group exhibited shorter escape latency on the fifth day compared with LPS-treated mice (*p* < 0.05, Figure 3(C)). Subsequently, the probe trial results showed that LPS-treated mice exhibited decreased platform crossing numbers (*p* < 0.05, Figure 3(D)) and less time spent on the platform quadrant (*p* < 0.01, Figure 3(D)) during the test period. However, *Astragalus* injection treatment increased the platform crossing numbers (*p* < 0.05, Figure 3(E)) and prolonged the swim time spent on the platform quadrant (*p* < 0.05, Figure 3(E)).

### *Astragalus* injection inhibited inflammatory cytokine synthesis and secretion

**Figure 4.**
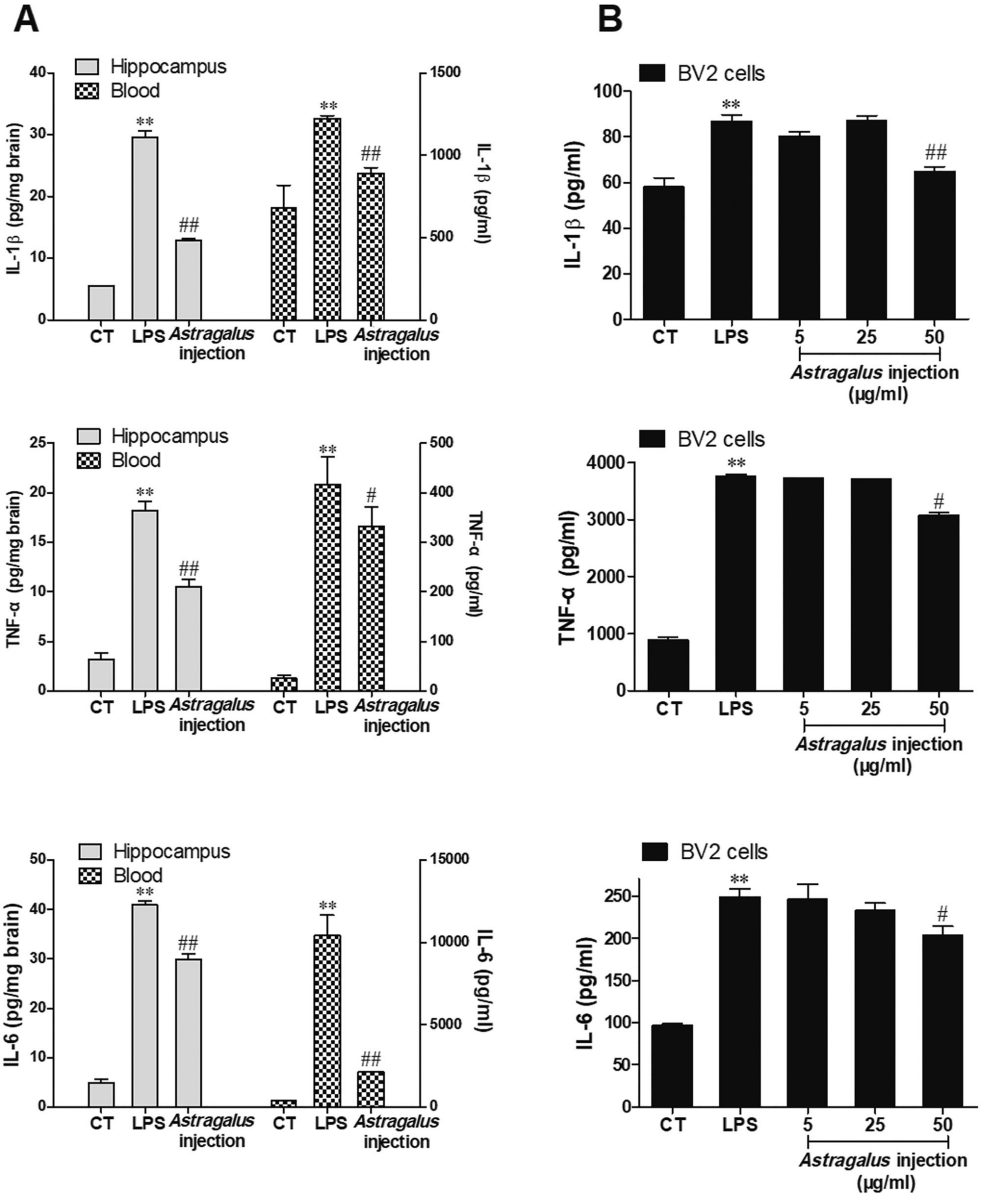
*Astragalus* injection improved LPS-induced IL-1β, TNF-α, and IL-6 protein synthesis and secretion. (A) LPS (2.5 mg/kg) was administered to mice for 3 days, followed by 5 days *Astragalus* injection (5 mg/kg per day, i.p.) treatment. The inflammatory cytokines in the mouse hippocampus (left, plot on the left *y*-axis) and blood (right, plot on the right *y*-axis) were evaluated. (B) Cultured BV2 cells were treated with LPS (1 µg/mL in the culture medium) for 2 h followed by 6 h of *Astragalus* injection co-incubation. Values are presented as means ± SD (*n* = 6). **p < 0.01 versus the control group; ^#^*p* < 0.05, ^##^*p* < 0.01, versus the LPS group. CT: control; LPS: lipopolysaccharide.

Growing evidence shows that inhibiting inflammatory cytokine (such as IL-1β) synthesis is a therapeutic strategy against LPS-induced cognitive dysfunction in mice (Liśkiewicz et al. 2019; Zhao et al. 2019; Jin et al. 2020). This study investigated the effects of *Astragalus* injection on inflammatory cytokine (IL-1β, TNF-α, and IL-6) protein synthesis *in vivo* and *in vitro*. For animal experiments, after 5 days of *Astragalus* injection treatment (5 mL/kg), the protein levels of inflammatory cytokines in the hippocampus and blood were evaluated by ELISA. As shown in Figure 4(A), LPS significantly induced the protein levels of IL-1β, TNF-α, and IL-6 in the hippocampus and blood compared with the control group. Besides, the protein levels of inflammatory cytokines in the hippocampus and blood significantly declined in the *Astragalus* injection group compared with the LPS group.

*Astragalus* injection was added after co-incubating LPS with BV2 microglial cells for 2 h for the *in vitro* study. Then, the secretion of IL-1β, TNF-α, and IL-6 proteins in the culture medium of BV2 cells was analyzed 6 h later via ELISA. Similar to the *in vivo* results, the protein levels of IL-1β, TNF-α, and IL-6 in the culture medium significantly increased after LPS stimulus (Figure 4(B)). Moreover, *Astragalus* injection could significantly decrease the IL-1β, TNF-α, and IL-6 secretion in a dose-dependent manner (Figure 4(B)).

### *Astragalus* injection overcame LPS-induced neuroinflammation and blood–brain barrier damage

**Figure 5.**
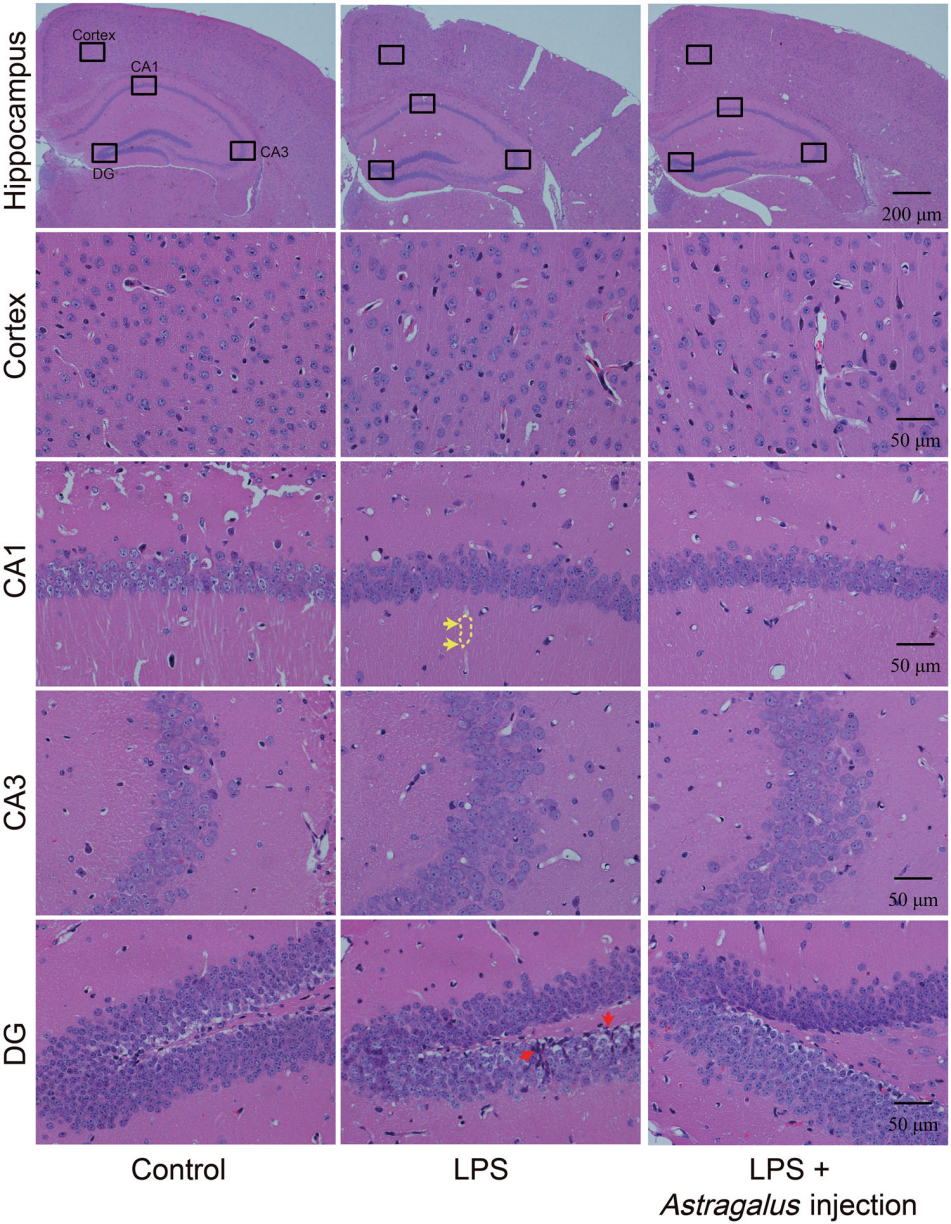
*Astragalus* injection improved the brain histopathological changes in mice (revealed by the H&E-stained brain sections). LPS: The mice received serial intraperitoneal injections of 2.5 mg/kg LPS dissolved in sterile normal saline. LPS + *Astragalus* injection: followed by administration of LPS, the mice were given 5 mL/kg *Astragalus* injection (i.p.), once a day for 5 days. Yellow arrows indicate the axon pale staining (marked with yellow dotted line); red arrows indicate the infiltration of lymphocytes. CA: cornu ammonis; CT: control; DG: dentate gyrus; LPS: lipopolysaccharide.

**Figure 6.**
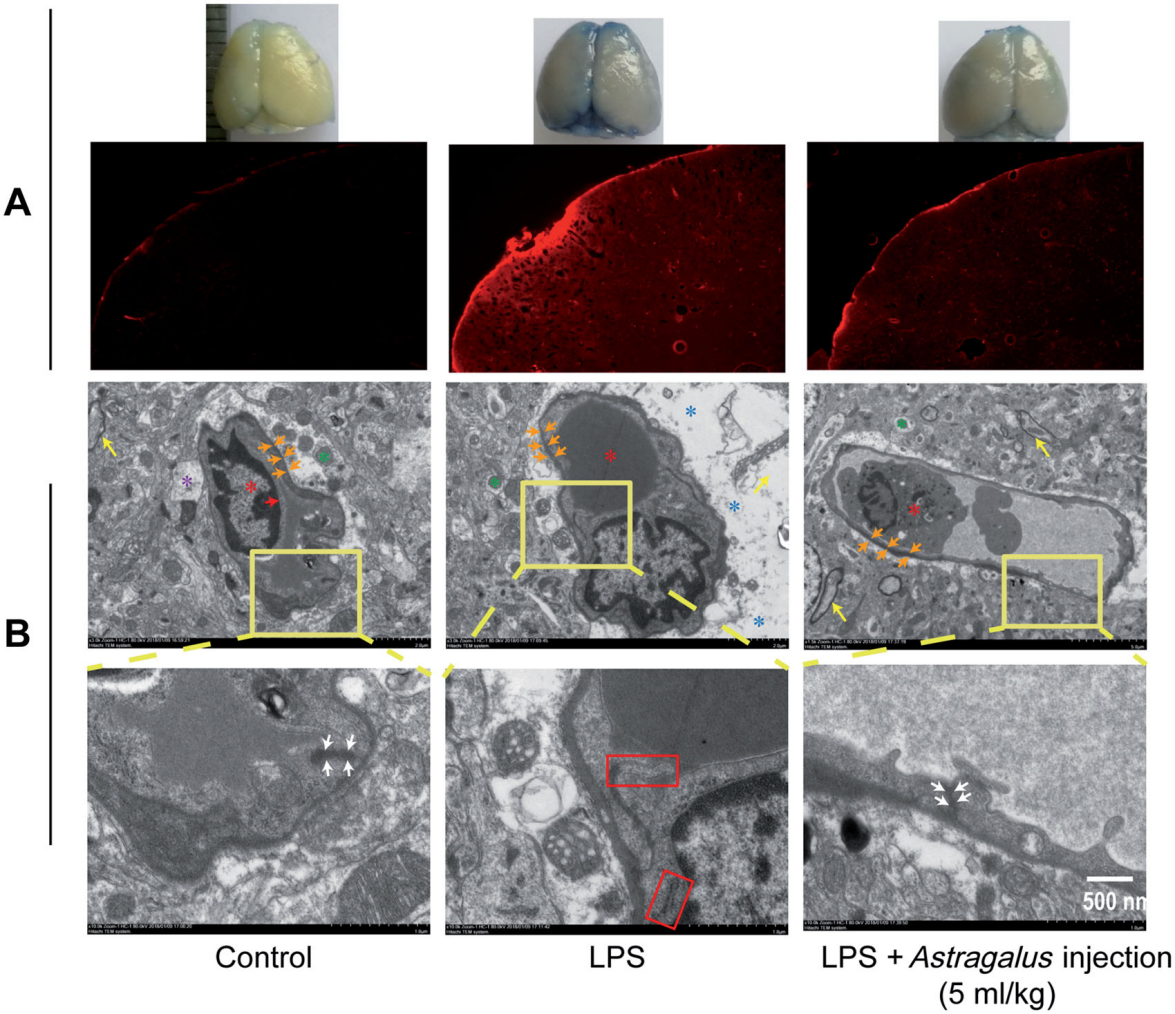
*Astragalus* injection improved LPS-induced BBB damage. (A) EB leakage in brain tissue. *The cerebral cortex was filled with robust fluorescent signals. (B) The ultrastructural changes in the BBB. Red asterisks indicate the vessels; red arrows indicate the endothelial cells; orange arrows indicate the basal lamina; purple asterisks indicate the astroglial end feet; green asterisks indicate the axon or dendrite; yellow arrows indicate the myelin sheath; blue asterisks indicate the swollen astroglial end feet; white arrows indicate the tight junction; and red panels indicate the detached tight junction. LPS: The mice received serial intraperitoneal injections of 2.5 mg/kg LPS dissolved in sterile normal saline. LPS + *Astragalus* injection: followed by administration of LPS, the mice were given 5 mL/kg *Astragalus* injection (ip), once a day for 5 days. CT: control; LPS: lipopolysaccharide.

Haematoxylin and eosin staining of brain tissue, Evans blue perfusion, and transmission electron microscopy analysis were conducted after 5 days of *Astragalus* injection treatment to further observe the effects of *Astragalus* injection on neuroinflammation and blood–brain barrier structure (Figures 5 and 6). The hippocampus H&E staining showed that LPS induced axon staining attenuated in the cornu ammonis (CA) 1 area (marked with yellow dotted line) and the infiltration of lymphocytes in the dentate gyrus (DG) area (Figure 5). *Astragalus* injection could ameliorate the aforementioned injury.

Evans blue is an azo dye that binds to albumin in blood and cannot traverse through normal BBB. Therefore, the fluorescence intensity of Evans blue in the brain reflects the damage status of BBB structure. The mouse brains were conspicuously stained in blue 24 h after 5 days of *Astragalus* injection treatment, and the whole cerebral cortex was filled with robust fluorescent signals (Figure 6(A)). *Astragalus* injection markedly attenuated the blue staining in the mouse brains, as reflected by the fluorescent intensity of Evans blue (Figure 6(A)). The ultrastructural changes in BBB were also examined. As shown in the control group, the BBB unit was composed of endothelial cells, basal lamina, pericytes, and astrocyte end feet (Figure 6(B)). After the LPS injury, the basement membrane was disrupted and tight junctions were damaged, in addition to evident swollen astrocyte end feet (Figure 6(B)). However, the basement membrane was preserved, and the tight junctions were restored in the *Astragalus* injection-treated group (Figure 6(B)).

### *Astragalus* injection improved the protein expression levels of tight junction proteins

The tight junction between brain microvascular endothelial cells is the fundamental structure of BBB, the first barrier to maintain homeostasis of the cerebral microenvironment. The tight junction is composed of transmembrane proteins (encompassing occludin, claudin, and junction adhesion molecules), cytoplasmic attachment proteins (encompassing zonula occludens (ZO)-1, −2, and −3 proteins), and cytoskeleton protein F-actin (Huber et al. 2001). The expression levels of these proteins are closely relevant to the BBB functions.

The results showed that *Astragalus* injection could restore LPS-induced tight junction damage in BBB. Subsequently, the levels of tight junction proteins levels were further evaluated via immunofluorescence staining. CD31, a surface protein, is expressed ubiquitously within the vascular compartment and is located mainly at junctions between adjacent cells (Lertkiatmongkol et al. 2016). Thus, it can be used as a marker for vascular or endothelial cells. The immunofluorescence double-labeling of CD31 and tight junction proteins (claudin-5, occludin, and ZO-1) was conducted to confirm the expression levels of tight junction proteins in microvascular endothelial cells.

**Figure 7.**
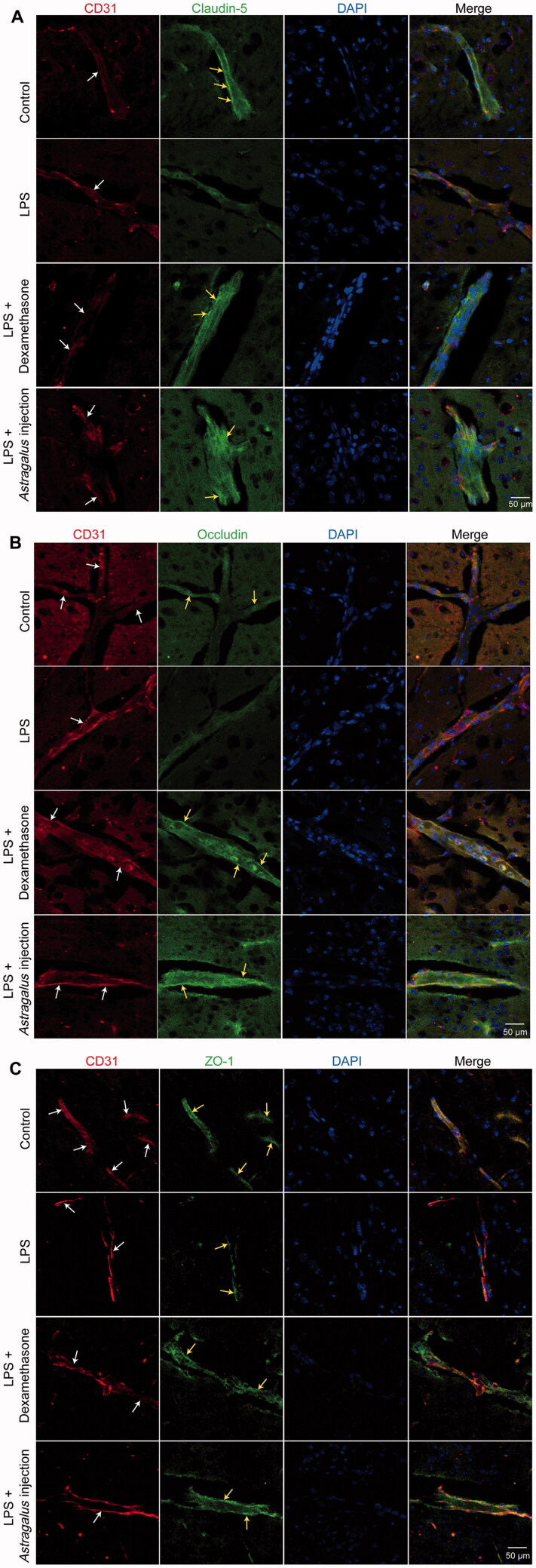
The effects of *Astragalus* injection on claudin-5 (A), occludin (B) and ZO-1 (C) expression detected by immunofluorescence staining. White arrows indicate the capillaries (CD31 positive staining indicated vascular endothelial cells); yellow arrows indicate the positive staining of claudin-5 (A), occludin (B) and ZO-1 (C). LPS: the mice received serial intraperitoneal injections of 2.5 mg/kg LPS dissolved in sterile normal saline. LPS + *Astragalus* injection: followed by administration of LPS, the mice were given 5 mL/kg *Astragalus* injection (i.p.), once a day for 5 days. CT: control; LPS: lipopolysaccharide.

The image from the control group reflected a high expression of claudin-5, occludin, and ZO-1 on microvascular endothelial cells (Figure 7). However, after LPS injury, the green fluorescence-labeled claudin-5, occludin, and ZO-1 were detected only in a few vascular cells (Figure 7). In addition, the green fluorescence of tight junction proteins (claudin-5, occludin, and ZO-1) were enhanced in the *Astragalus* injection group (Figure 7).

### *Astragalus* injection prevented neurodegeneration in the hippocampus

**Figure 8.**
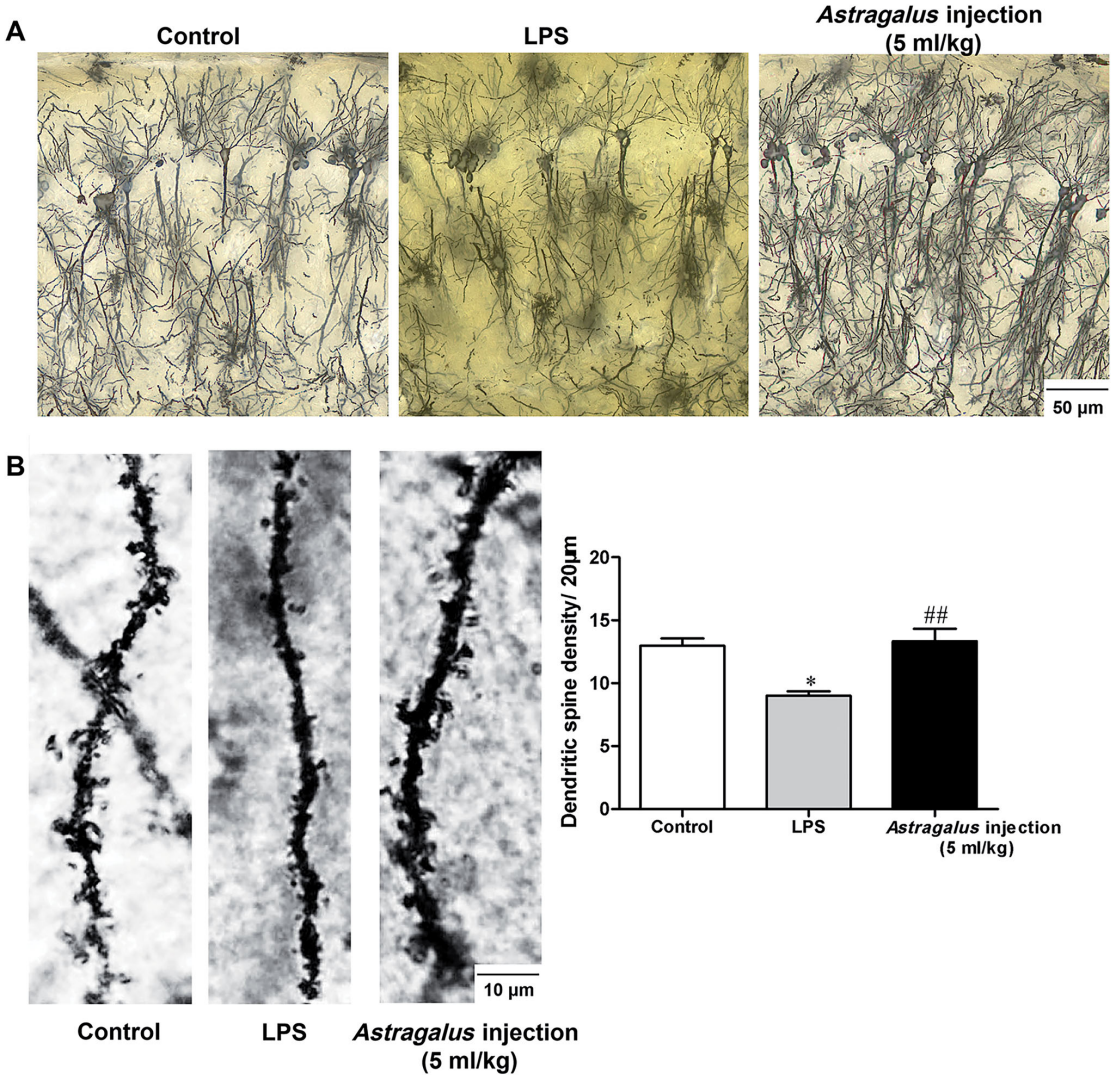
*Astragalus* injection prevented the neurodegeneration in the hippocampus of LPS-exposed mice. (A) Golgi silver-stained neurons in the hippocampal CA1 subfield. Bar = 50 μm. (B) Representative dendritic spine and dendritic spine density in the CA1 hippocampal region (*n* = 9–13). Bar = 10 μm. *p < 0.05 versus the control group; ^##^*p* < 0.01 versus the LPS group. CT: control; LPS: lipopolysaccharide.

Neurodegeneration contributes to the deterioration of memory performance. Behavioural tests demonstrated that *Astragalus* injection could improve recognition memory in mice. Then, Golgi staining of the hippocampus was conducted after the behavioural tests to confirm the role of *Astragalus* injection in preventing neurodegeneration of LPS-treated mice. In serial coronal sections of the hippocampus, the neurons had decreased complexity of dendritic trees, less dendritic branching, and reduced branch length in the hippocampal CA1 subfield in the LPS group compared with the control group, which was reversed by *Astragalus* injection treatment (Figure 8(A)). In addition, significantly decreased dendritic spine density was observed in LPS-treated mice (*p* < 0.05, Figure 8(B)), which was restored by *Astragalus* injection (*p* < 0.01, Figure 8(B)).

### *Astragalus* injection activation of TrkB pathway

#### Molecular docking results of *Astragalus* injection main active components to TrkB

TrkB is a neurotrophin receptor that binds to BDNF, and one of its downstream signalling transduction pathway is CaMKIIα/CREB. The CaMKIIα/CREB/BDNF pathway is coupled with the cognitive function (Kozisek et al. 2008; Tang et al. 2019; Esvald et al. 2020). Based on the analysis results of *Astragalus* injection, astragaloside, formononetin, formononetin-7-*O*-glucoside, and calycosin were selected to further evaluate their interaction with TrkB via molecular docking test. Results showed that the estimated binding energy values were −5.0, −6.9, −6.3, and −7.0 kcal/mol for astragaloside, formononetin, formononetin-7-*O*-glucoside, and calycosin, respectively.

**Figure 9.**
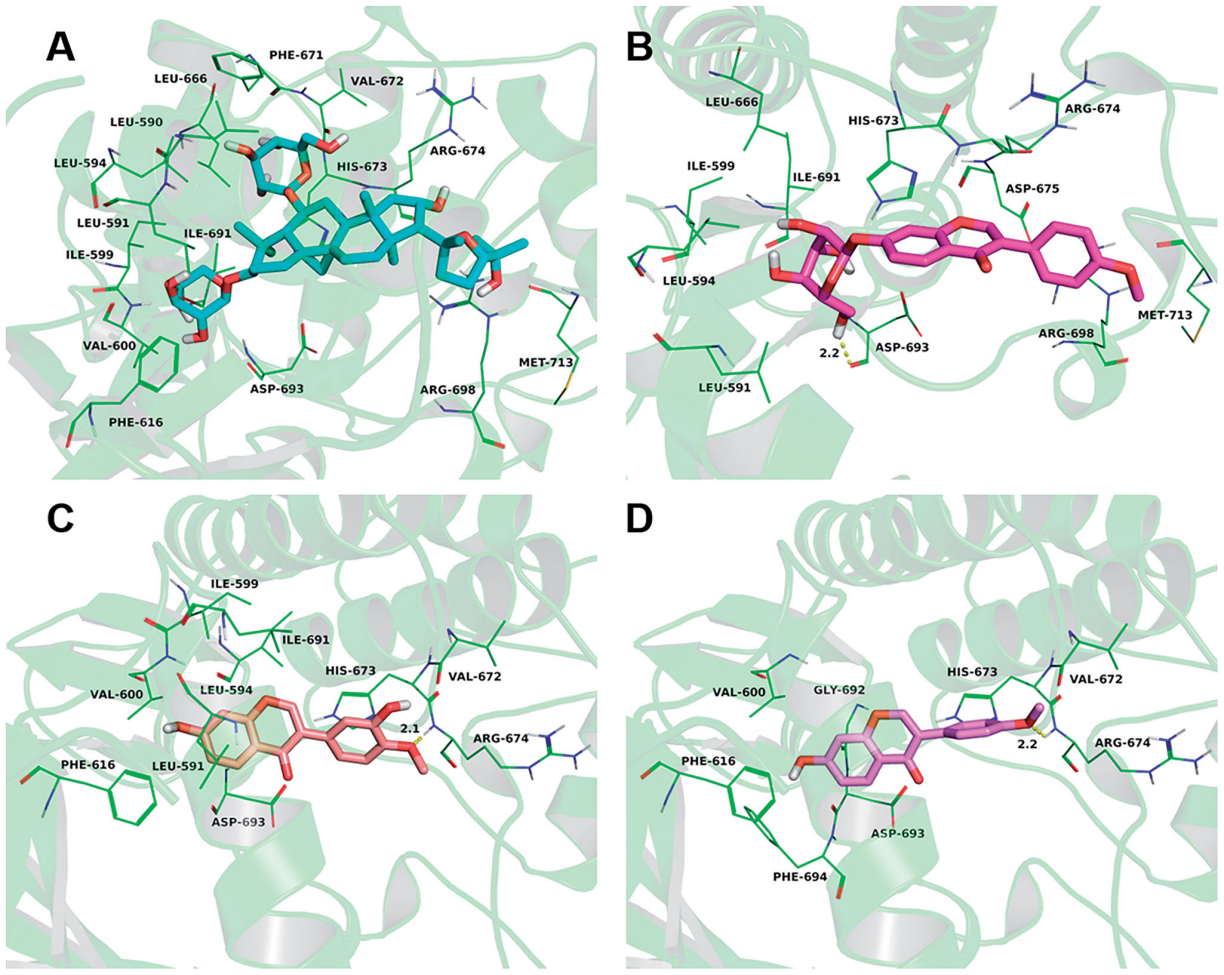
*Astragalus* injection four main active components were docked into the binding site of the *Mus musculus* TrkB (detailed view). The representative binding residues within 4.0 Å of this substrate were shown in green lines; (A) the astragaloside was represented with cyan sticks. (B) The formononetin was represented with violet sticks. (C) The formononetin-7-*O*-glucoside was represented with rose red sticks. (D) The catalpol was represented with orange sticks.

The theoretical binding mode of the test compounds in the binding site of the TrkB was illustrated in Figure 9. For astragaloside, it stretched into the hydrophobic pocket that consisted of Leu-590, Leu-591, Leu-594, Ile-599, Val-600, Phe-616, Leu-666, Phe-671, Val-672, and Ile-691, forming strong hydrophobic bindings (Figure 9(A)). For formononetin, it formed the cation-π interaction and anion-π interaction with the residues Arg-674 and Asp-693, respectively. Importantly, one key hydrogen bond interaction was observed between the formononetin and the residue Arg-674 (bond length: 2.2 Å), which was the main interaction between the formononetin and the TrkB (Figure 9(B)). For formononetin-7-*O*-glucoside, one key hydrogen bond interaction was observed between it and the residue Asp-693 (bond length: 2.2 Å), which was the main interaction between the it and the TrkB (Figure 9(C)). For calycosin, it stretched into the hydrophobic pocket that consisted of Leu-591, Leu-594, Ile-599, Val-600, Phe-616, Val-672, and Ile-691, forming strong hydrophobic bindings (Figure 9(D)).

### *Astragalus* injection prevented the reduction of TrkB/CaMKIIα/CREB/BDNF pathway-associated proteins

**Figure 10.**
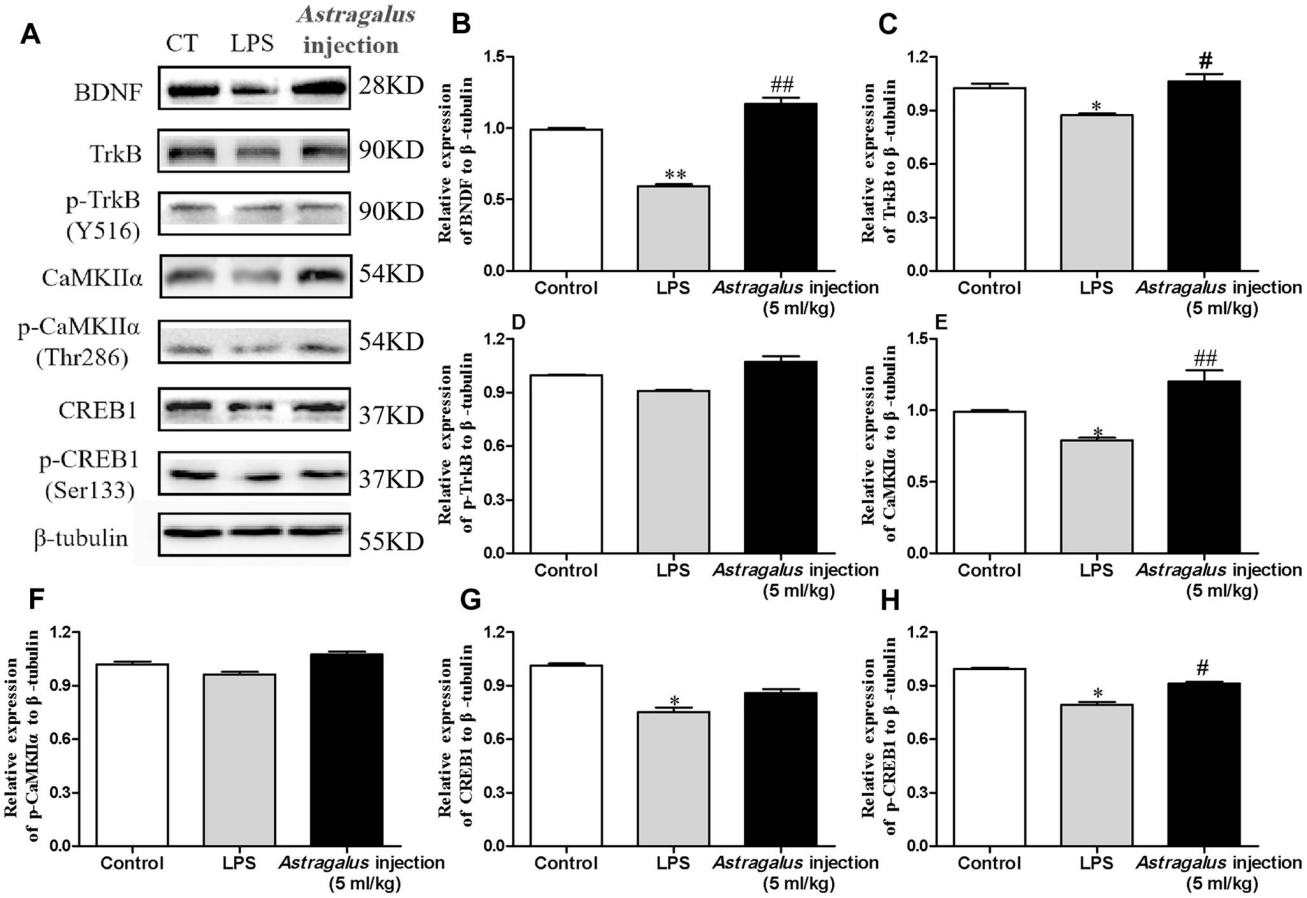
Effect of *Astragalus* injection on the BDNF/TrkB/CaMKIIα/CREB pathway in the hippocampus. (A) Western blot bands for hippocampal BDNF, TrkB, p-TrkB(Y516), CaMKIIα, p-CaMKIIα (Thr286), CREB1, and p-CREB1 (Ser133). (B–H) Quantification of hippocampal BDNF, TrkB, p-TrkB(Y516), CaMKIIα, p-CaMKIIα (Thr286), CREB1, and p-CREB1 (Ser133) western blot analyses in A. Values are presented as means ± SD (*n* = 6). *p < 0.05 versus the control group; ^#^*p* < 0.05, ^##^*p* < 0.01, versus the LPS group. CT: control; LPS: lipopolysaccharide.

The molecular docking results showed that the main active components in *Astragalus* injection could interact withTrkB. Subsequently, the protein expression of BDNF, TrkB, p-TrkB(Y516), CaMKIIα, p-CaMKIIα (Thr286), CREB1, and p-CREB1 (Ser133) in the hippocampus was examined after behavioural tests using western blot analysis to confirm an association between improving recognition memory effects of *Astragalus* injection and tyrosine kinase receptor B (TrkB)/CaMKIIα/CREB/BDNF pathway. The results showed that the LPS-treated mice had lower levels of hippocampal BDNF, TrkB, CaMKIIα, CREB1, and p-CREB1 (Ser133) expression compared with the control group (*p* < 0.05, Figure 10). However, the treatment with *Astragalus* injection showed positive effects and elevated the expression levels of the aforementioned proteins (*p* < 0.05, Figure 10).

## Discussion

The present study showed that LPS-induced sepsis in mice not only led to systemic inflammatory and BBB dysfunction but also caused long-term cognitive behavioural deficits. Further studies showed that *Astragalus* injection treatment could reduce LPS-induced neuroinflammation. Additionally, continuous repeated treatment using *Astragalus* injection could reverse BBB dysfunction, prevent neurodegeneration, and upregulate the BDNF–CREB pathway during LPS-induced sepsis, ultimately preventing the development of cognitive decline. Taken together, the data suggested that *Astragalus* injection could be a valuable preventive and therapeutic strategy for sepsis survivors in clinical settings via various therapeutic targets and mechanisms of action.

Accumulating evidence proves that inflammation and neurodegeneration are related events. A large number of reactive oxygen species, cytokines, and nitric oxide are produced when leukocytes (especially macrophages) respond to Gram-negative bacteria infection in the pathogenesis of septic shock (Henry et al. 2009; Hoogland et al. 2018). Inflammatory cytokines released from macrophages directly activate microglia in the brain, which in turn produce pro-inflammatory cytokines and result in neuron injury (Michels et al. 2014; Hoogland et al. 2015, 2018). A recent study indicated that the aged mice had decreased methylation of the IL-1β gene promoter in microglia, which was associated with increased intracellular IL-1β production, as well as prolonged sickness behaviour after LPS injury (Matt et al. 2016). Another study also showed that minocycline could improve long-term cognitive impairment in sepsis survivors via decreasing acute brain oxidative damage and inflammation (Hoshino et al. 2017) Similarly, Reis et al. (2017) reported that statins could prevent cognitive impairment after sepsis by reverting neuroinflammation. In line with the previous findings, the present study showed a cause-and-effect relationship between *Astragalus* injection decreasing the protein levels of TNF-α, IL-1β, and IL-6 and ameliorating neurodegeneration in the hippocampus. Meanwhile, the treatment of *Astragalus* injection decreased the protein levels of TNF-α, IL-1β, and IL-6 in the blood and hippocampus after LPS stimulation. Hence, it was suggested that *Astragalus* injection could be a potential preventive strategy against sepsis.

BBB is a major internal barrier between peripheral circulation and the central nervous system. It strictly restricts the transport of substances that are neurotoxic to protect the central nervous system. It comprises brain microvascular endothelial cells, pericytes, astrocytic end feet, basal lamina, neurons, and microglia (Huber et al. 2001). Among them, the tight junction between brain microvascular endothelial cells is the fundamental structure of BBB, the first barrier to maintain homeostasis of the cerebral microenvironment. LPS-induced inflammation could disrupt the integrity and function of BBB (Banks et al. 2015; Danielski et al. 2018). Nishioku et al. (2009) reported that pericyte detachment and microglial activation might be involved in the mediation of LPS-induced BBB disruption due to inflammatory responses in the damaged brain. A previous *in vitro* study revealed that LPS destroyed the integrity of mouse brain microvascular endothelial cells via disaggregating cytoskeleton actin and downregulating tight junctional proteins, such as claudin-5, occludin, and ZO-1, as well as increasing the secretion of endothelin-1 and inflammatory cytokines (Feng et al. 2018). Consistent with previous results, the present study showed disrupted ultrastructure of BBB and downregulation of tight junctional proteins in mice after the LPS injury. However, *Astragalus* injection could reverse this injury, suggesting that the translocation of inflammatory cytokines into BBB was inhibited. Thus, the microglia activation and related neuron injury were ameliorated partly.

A recent review summarized that BDNF was involved in the formation of different types of memories and also critical for maintaining the long-lasting storage of information in the hippocampus (Kozisek et al. 2008). It was similar to another report showing that the hippocampus-specific deletion of BDNF in adult mice impaired spatial memory and caused extinction of aversive memories (Heldt et al. 2007). BDNF binds to its high-affinity receptor TrkB, triggering the activation of one or more of three major signalling pathways, such as CaMKIIα, phosphatidylinositol 3-kinase (PI3K), phospholipase C gamma (PLC-γ), and extracellular signal-regulated kinase 1/2 (ERK1/2), regulating certain forms of synaptic plasticity, including long-term potentiation and affecting synaptic transmission (Koike et al. 2013; Adachi et al. 2017; Xenos et al. 2018). The molecular docking results indicated that *Astragalus* injection could upregulate BDNF expression and activate its downstream pathways TrkB/CaMKIIα/CREB, thus produced neuroprotection with resulting improvement in memory function.

In addition, previous studies showed that Astragali Radix polysaccharides could improve impaired learning and memory functions in aged rats by upregulating the activity of hippocampal CREB/BDNF cascade (Yao et al. 2014). Meanwhile, a chemical analysis for *Astragalus* injection reflected that it contains polysaccharides with the average molecular weight of polysaccharides was between 7684 and 108,846 (Jian et al. 2010). However, whether the Astragali Radix polysaccharides in *Astragalus* injection also contributed to the protection effects on post-sepsis cognitive impairment is still unknown. Further studies should be conducted to verify the hypothesis.

## Conclusions

The present study suggested that *Astragalus* injection could be used in the prevention and treatment of post-sepsis cognitive impairment via inhibition of inflammatory processes, maintenance of BBB integrity, and upregulation of neurotrophic factor in different stages of post-sepsis sequelae.

## Data Availability

The datasets used and/or analyzed during the current study are available from the corresponding author on reasonable request.
